# Changes in bone quality after switching from a TDF to a TAF based ART: A pilot randomized study

**DOI:** 10.3389/fendo.2023.1076739

**Published:** 2023-03-27

**Authors:** Jade Soldado-Folgado, Oriol Rins-Lozano, Itziar Arrieta-Aldea, Alicia Gonzále-Mena, Esperanza Cañas-Ruano, Hernando Knobel, Natalia Garcia-Giralt, Robert Güerri-Fernández

**Affiliations:** ^1^ Departament de Medicina, Universitat Autònoma de Barcelona, Barcelona, Spain; ^2^ Department of Internal Medicine, Hospital del Mar Institute of Medical Research (IMIM), Barcelona, Spain; ^3^ Department of Medicine and Life Sciences (MELIS), University Pompeu Fabra, Barcelona, Spain; ^4^ Department of Infectious Diseases, Hospital del Mar Institute of Medical Research (IMIM), Barcelona, Spain; ^5^ Instituto de Salud Carlos III, Centro de Investigación Biomédica en Red Fragilidad y Envejecimiento Saludable (CIBERFES), Madrid, Spain; ^6^ Instituto de Salud Carlos III, Centro de Investigación Biomédica en Red Enfermedades infecciosas (CIBERINFEC), Madrid, Spain

**Keywords:** bone quality, microindentation, antiretroviral therapy, HIV infection, fracture risk

## Abstract

**Background:**

The impact of tenofovir disoproxil fumarate (TDF) antiretroviral (ART) regimens on bone health has been characterized mostly by bone mineral density (BMD), but recently also by bone quality (BQ). The aim of this pilot study is to assess the changes in BMD and BQ after switch from TDF to tenofovir alafenamide (TAF) ART.

**Methods:**

HIV individuals receiving TDF-based ART were randomized to switch to Bictegravir-TAF-Emtricitabine or to remain in the same regimen. At baseline and 24-weeks after randomization, participants underwent bone mineral density (BMD) by DXA and BQ assessment using bone microindentation, a validated technique that measures bone tissue quality expressed as bone material strength index (BMSi). A panel of plasma bone turnover biomarkers were measured by ELISA at the same time-points. Values are expressed as median [interquartile range] and non-parametric tests were used where appropriate.

**Results:**

A total of 24 HIV individuals were included in the study, 19 of which were men (80%). Median age at baseline was 43 years (IQR 38-54). Half of individuals were allocated in the TDF group while the other half changed to TAF treatment. No differences at baseline between both groups were detected in any parameter. Non-significant changes nor in lumbar or femoral BMD at week 24 was found in any regimen. In contrast, there was an increase in BMSi in the TAF arm at 24 weeks, and thus an improvement in BQ[81.6 (79-83) to 86 (80-88) (+5.1%);p=0.041], whereas the TDF arm remained stable from 82 (76-85) at baseline to 82 (73-83);p=0.812. Hence, at week 24 there were significant differences in BQ between arms (p=0.049). A reduction in bone formation markers was found at week 24 in both regimens: N-terminal propeptide of type-1 collagen decreased a 20% (-35 - -0.6); p=0.031 with TAF and -16% (-25 - -5); p=0.032 with TDF. Also a decrease in bone resorption marker C-telopeptide with TAF was detected [-10% (-19 - -5);p=0.028] but not with TDF (p=0.232), suggesting a less metabolically active bone after switching to TAF.

**Conclusion:**

A bone quality improvement was found after switching from a TDF to a TAF based ART independently of BMD, suggesting that the bone health benefits of TAF may extend beyond BMD. Future research should be directed to confirm these findings and to identify the underlying mechanisms of ART related bone toxicity.

## 1 Introduction

People living with HIV (PLWHIV) experience up to 4-fold higher annual rates of fragility fractures than the general population (1–3). As PLWH live longer through effective antiretroviral therapy (ART), fracture rates are expected to further increase in the future.

In clinical trials in PLWHIV, tenofovir disoproxil fumarate (TDF) was associated with a greater decrease in bone mineral density (BMD) and an increase in biochemical markers of bone metabolism, suggesting increased bone turnover relative to comparators. Whether these changes in BMD were associated with an increased risk of fractures has been controversial. However, some cohort studies (2, 4, p.) suggest that having lower BMD is associated with a higher risk of fractures,. Tenofovir alafenamide Fumarate-TAF- has shown a better profile of bone safety when compared with TDF. A prior study showed that people with low bone mineral density who switched from TDF to TAF experienced improvements in bone health such as a reduced risk of osteoporosis.

While several studies have emphasized an increased fracture incidence in PLWHIV (2, 3), this increased fracture incidence is not fully explained by differences in bone mineral density (BMD) between PLWHIV individuals and healthy controls. An emerging explanation for this paradox is that HIV infection and treatment are associated with changes in bone quality. Changes affecting the microarchitecture as well as the composition of the bone matrix and non-collagenous proteins (5) can affect bone quality and, consequently, on a higher risk of fracture. Microindentation is a technique cleared by the FDA that allows direct evaluation of the quality of bone material, encompassing these material-dependent elements not captured by BMD.

Microindentation allows detecting changes in bone quality much earlier than BMD (6). For all these reasons, the present study aims to assess the changes in bone quality in a group of people living with HIV who change from a TDF-based therapy to a TAF-based therapy.

## 2 Methods

### 2.1 Population and study design

This is a pilot open-label, randomized, unicenter, 24-week clinical trial conducted that was carried out in a university hospital in Barcelona, Spain between July 2019 to June 2020. This study enrolled HIV-1- positive adults who were virologically suppressed on any TDF (tenofovir disoproxil fumarate plus emtricitabine plus a third drug) based approved 3-drug regimen for at least 48 weeks and were randomly assigned (1:1) to receive bictegravir 50mg plus tenofovir alafenamide 25 mg and emtricitabine 200mg (TAF) for 24 weeks or continue their baseline disoproxil fumarate based regimens.

Inclusion criteria were age ≥18 years, ≥2 HIV-1 RNA measurements <50 copies/mL within 48 weeks of study entry, and a screening HIV-1 RNA <20 copies/mL. We considered as ineligible a history of virologic failure (VF) after 1 year of treatment, pretreatment reverse transcriptase (RT) resistance mutation, or known integrase resistance mutations, and those individuals who had previously received treatments that might have affected the bone quality, such as systemic glucocorticoids or anti-osteoporotic medications. We also excluded individuals who had previously been diagnosed with chronic kidney disease, chronic endocrine conditions, malabsorption syndrome, advanced liver disease, neoplasia, and bone diseases.

### 2.2 Procedures

After screening of inclusion/exclusion criteria, study visits occurred at day 1 and week 24. At day 1 participants underwent a baseline-randomization visit where clinical history was recorded, and a general physical examination was performed. Lateral spinal X-rays were taken and assessed by two independent observers to detect any vertebral fractures, defined as deformities of grade I or above (a loss of >20% of vertebral height).

#### 2.2.1 Bone mineral density

BMD was measured at the lumbar spine and hip using dual energy x-ray absorptiometry (DXA) (Hologic QDR 4500 SR, Hologic, Inc., Bedford, MA, USA). Values were expressed as *g/cm^2^* of mineral content. The coefficient of variation for the DXA measurements was 1.8%

#### 2.2.2 Bone microindentation measurements

Bone microindentation was measured using an Osteoprobe instrument (Active Life Scientific, Santa Barbara, CA, USA) according to a protocol previously described (7). In brief the testing takes place on the anterior face of the mid-tibia under local anesthesia. A needle applied through the skin is pushed into the bone surface with a force of 30 N during less than a millisecond creating an indentation, or microfracture, on the bone surface. The software registers the distance from the needle tip right before impact and right after, a distance called the total indentation. Repeated measurements in the same area are taken and right after, five measurements are performed on a piece of Poly-methyl-methacrylate (PMMA). Bone microindentation yields a dimensionless quantifiable parameter called bone material strength index (BMSi), which is positively correlated with bone tissue quality. The BMSi is calculated as 100 times the ratio of the mean total indentation in the PMMA and the tibia.

To minimize interobserver variation, all measurements for this study were taken by the same investigator (RGF) that was blinded for arm of treatment. As previously described, the microindentation procedure is minimally invasive, safe, painless, and takes less than 5 minutes and the software provides results immediately. Contraindications for this technique included local skin infection, significant local oedema, and/or thick subcutaneous adipose tissue at the site of indentation.

#### 2.2.3 Laboratory assays

Chemiluminescent immunoassays (CLIA) was used to determine bone turnover markers and other bone specific parameters (based on fasting blood samples). Each immunoassay had an inter-assay coefficient of variation, iCV, of 10%. Specifically, we measured levels of intact parathyroid hormone (iPTH) (Siemens), bone alkaline phosphatase (Roche Diagnostics), amino propeptide of type I collagen (P1NP, Roche Diagnostics), collagen type I C-telopeptide (CTX, Roche Diagnostics), serum 25-hydroxyvitamin D_3_ (Roche Diagnostics).

### 2.3 Study outcomes

The main outcome was the mean percentage change in bone tissue quality measuring the Bone Material Strenght index (BMSi) by microindentation at 24-weeks post randomization. Secondary endpoints included the change in spine and hip bone mineral density (BMD), the change in CD4 cell count, and the mean percentage change in bone turnover markers from baseline to week 24

#### 2.3.1 Statistical methods

Sample size was calculated according to previous publications (8–10). Accepting an alpha risk of 0.05 and a beta risk of 0.2 in a two-sided test, 12 participants in each arm were needed to recognize a difference greater than or equal to 5 BMSi units as statistically significant. The standard deviation is assumed to be 4. A drop-out rate of 10% was anticipated.

Categorical variables were summarized using frequencies and percentage. Continuous variables were summarized using the median and interquartile range (IQR). Change in bone health parameters post switch to TAF-containing ART was assessed using Wilcoxon rank sum testing. And the Mann-Whithney U test was used to compared both arms.

Correlation between changes in bone quality, weight or body mass index were studied using Spearman’s correlation test.

All analyses were conducted using Stata 13.1 (StataCorp, College Station, Texas, USA).

The trial is registered with EUDRAT (num 2018-004499-36).

The institutional review board approved this study, and each participant provided informed consent.

## 3 Results

### 3.1 Patient characteristics

Twenty-four HIV individuals were included (**Table 1**). The median age was 45 years (IQR 38-54) and 19 (80%) were male. Regarding the immune status, the median CD4 T-cell nadir count was 388 cells per ml (IQR 225-423), the median current CD4 T-cell count was 603 cells per ml (IQR 507-789) and the median CD4/CD8 ratio was 1.01 (IQR 0.74-1.23). All individuals reported a good adherence to ART with viral load below 19 copies per ml (**Table 1**).

**Table 1:** Patient characteristics and bone health parameters before and after switch.

	*TDF arm*		*p-value^1^*	*TAF arm*		*p-value^2^*
	*Baseline*	*24-weeks*		*Baseline*	*TAF arm 24-weeks*	
Cohort	N=12	N=12		N=12	N=12	
Age, median years (IQR)	46 (40-53)			44 (36-48)		
Male, n(%)	10 (83%)			9 (75%)		
Smoker, n(%)	2 (14%)			3 (25%)		
*Weight (Kg)*	76.9 (71-85)	77 (67-88)	0.594	75.3 (70-84)	77 (73 – 89)	0.031
*Body Mass Index (BMI)*	25.3 (24-27)	26.1 (22-27)	0.594	24.6 (23-29)	27.1 (23.9-29.9)	0.026
Regimen at baseline						
Rilpivirine	4 (34%)			6 (50%)		
Elvitegravir/cobicistat	4 (34%)			5 (41%)		
Efavirenz	1 (9%)			1 (9%)		
Boosted Darunavir	2 (14%)			0		
Raltegravir	1 (9%)			0		
Bone Parameters						
BMSi	82.35 (76-85)	82 (73.5-83)	0.812	81.6 (79-83)	86 (80-88)	0.041
Lumbar spine BMD *(g/cm^2^)*	0.985 (0.804-1.042)	0.991(0.811-1.042)	0.552	0.981(0.851-1.036)	0.979 (0.863-1.041)	0.504
Femoral neck BMD *(g/cm^2^)*	0.739 (0.673-0.892)	0.794 (0.689-0.893)	0.109	0.792(0.723-0.830	0.791 (0.668-0.824)	0.929
T-score spine	-0.9 (-2.2 - -0.4)	-0.9 (-2.1 - -0.3)	0.978	-1 (-2 - -0.4)	-1 (-1.9 – 0)	0.367
T-score femoral neck	-1.3 (-1.7 - -0.2)	-1(-1.6 – 0.1)	0.067	-0.75(-1.35 - -0.6)	-0.8(-1.9 - -0.5)	0.836
Bone Metabolism Markers						
P1NP (ng/ml)	54.9 (46-100)	46 (37-69)	0.031	59.3 (46-73)	47 (39-59)	0.032
CTX (ng/ml)	0.351 (0.317-0.427)	0.441 (0.314-0.712)	0.916	0.362 (0.264-0.556)	0.355 (0.230-0.459)	0.028
Bone Alkaline Phosphatase (µg/ml)	17 (14-21)	15.8 (14.6-17.9)	0.109	14.5 (12.4-17.4)	10.5 (8.9-14.2)	0.202
iPTH(pg/ml)	33 (25-47)	34 (24-48)	0.735	35(26-43)	38(26-44)	0.342
25OH Vitamin D (ng/ml)	26.3 (26-31)	20 (17-24)	0.009	25 (11-34)	16 (6-28)	0.003
HIV specific parameters						
CD4+ T-cell/ml median (IQR)	584 (574-892)	631 (602-738)	0.327	609 (390-751)	740 (412-1093)	0.012
CD4/CD8 ratio median (IQR)	1.06 (0.986-1.224)	0.928 (0.798-1.235)	0.674	0.851 (0.595-1.230)	0.761 (0.593-1.104)	0.139
Viral load median (IQR)	19 (9.5-19)	19 (0-19)	0.373	19 (19-19)	19 (0-19)	0.973

Results are shown as median values (IQR), unless indicated otherwise. ^1^ corresponds to the p-value when comparing TDF arm 24-week to baseline. ^2^ corresponds to the p-value when comparing TAF arm 24 week to baseline. Bold font represents significant differences after 24 weeks.

Patients were randomly separated in 1:1 proportion in two arms according to ART regimen and all of them completed the 24-week follow-up visit. No differences were detected in any baseline characteristic between arms.

### 3.2 The switch of TDF to TAF elicited changes regarding bone quality

**Figure 1:**
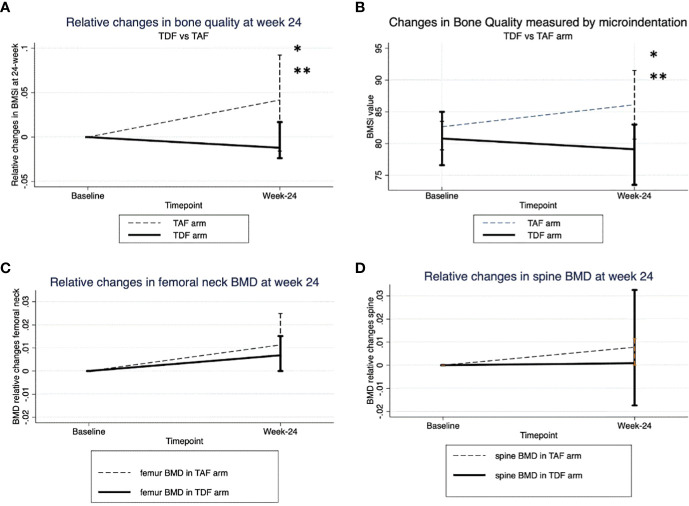
**(A)** Absolute changes in BMSi between two arms. *p-value at week -24 respect to baseline in TAF arm. **p-value at week-24 between TDF and TAF arms. **(B)** Relative changes in bone quality. **(C)** Relative changes in Bone mineral density at femoral neck. **(D)** Relative changes in Bone mineral density at spine neck.

From baseline to 24-week after randomization we observed a significant increase in bone tissue quality measured by microindentation only in the TAF arm [81.6 (79-83) to 86 (80-88)] (mean percentage change +5.1%); p=0.041, whereas BMSi values remained stable in the TDF group 82.35 (76-85) to 82 (73.5-83) (mean percentage change -0.05%); p=0.812 (**Table 1**). This result was indicative of improved bone tissue quality after switching to TAF (p=0.041). Moreover, there were significant differences in BQ between arms at week 24 (p=0.049). (**Figures 1A, B**). In contrast, no significative change was detected in BMD values at any arm (**Table 1**) (**Figures 1C, D**).

### 3.3 Bone turnover markers

The bone formation marker N-terminal propeptide of type-1 collagen (P1NP) was significantly decreased in both arms at week 24 (**Table 1**) with a mean percentage change of -16%; p=0.031 in the TDF arm, and a mean percentage change of -20%; p=0.032 in the TAF arm. Moreover, a significant decrease in bone resorption marker C-telopeptide was detected in the TAF arm with a mean percentage change of -10% (-19 - -5);p=0.028 but not with TDF; p=0.232, suggesting a less metabolically active bone after switching to TAF. No differences were found in Bone Alkaline Phosphatase neither in the TDF or TAF groups.

In individuals allocated in the TAF arm we detected a positive correlation between changes in weight and changes in bone quality BMSi (Spearman Rho’s 0.510;p=0.021) after 24 weeks of follow up.

Significant changes in 25-OH vitamin D_3_ levels were also detected in both arms, reflecting the seasonal variation of this hormone (**Table 1**).

## 4 Discussion

We report a comprehensive assessment of bone health in treated PLWHIV after a randomized pilot study with switching from a TDF-based regimen to a TAF-based regimen. In this study individuals who switched to a TAF-based regimen experienced a significant improvement in bone tissue quality measured by bone microindentation at week 24, whereas no changes in bone mineral density were found.

Until now, the only way to measure the mechanical resistance of bone was through *ex vivo* techniques, which made it difficult to apply in the clinical setting. Microindentation allows direct study *in vivo* of the resistance of bone tissue to an impact of known and controlled force. Compared with other techniques, microindentation has shown earlier detection of changes in bone quality. For instance, Mellibovsky et al. detected changes in bone quality just 7 weeks after starting treatment with glucocorticoids, while no changes in bone mineral density had yet been detected (6). Our group has reported altered bone quality in PLWHIV compared to HIV negative individuals, while there were no differences in BMD (7).

TDF has been repeatedly associated with its toxic effect on bone. TDF directly interferes with bone homeostasis through the reduction of extracellular adenosine levels, mediated by inhibition of ATP release from cells (11, p.; 12). As a result, there is a stimulation of osteoclast differentiation and osteoblast inhibition, with increased bone resorption. In addition, TDF interferes with the binding of calcidiol with its carrier protein (DBP, vitamin D binding protein) reducing its availability for the production of 1,25-dihydroxyvitamin D (calcitriol, the active form of vitamin D) in the kidney (13). Reduced calcitriol will also result in less calcium and phosphorus being absorbed in the intestine, which will promote the emergence of secondary hyperparathyroidism and increased bone resorption. However, although we found changes in vitamin D levels, no cases of secondary hyperparathyroidism were found among participants.

Interestingly, we found in a previous study that starting antiretroviral treatment improved bone quality despite the fact that bone mineral density decreased in the first weeks of treatment (9, 10) likely as a consequence of the control of the viral replication along with the immune reconstitution. In this study PLWHIV under chronic treatment with a TDF-based therapy experienced a median increase of 5% in BMSi values, showing an improvement in bone quality. Likely showing a better profile of TAF in bone quality compared to TDF.

In the present study, individuals under TDF regimen at the timepoint of 24 weeks after randomization remained stable regarding bone strength parameters.

Tenofovir alafenamide is the tenofovir prodrug. TAF is mostly metabolized intracellularly by cathepsin A to tenofovir, whereas TDF is hydrolyzed by intestine and plasma esterases to tenofovir (14). As a result, when compared to TDF, the pharmacokinetics of TAF enabled a reduction of nearly 91% in plasma concentrations of the active metabolite of tenofovir, lowering the exposure of the kidney and bone to the medication. This explains the different behavior of bone properties when exposed to both drugs.

TAF-containing regimens showed significantly lower decrease in glomerular filtration rate, less proteinuria and less reduction in BMD in comparison with those receiving TDF-containing regimens. In addition, patients on TDF who switched to TAF had increased BMD (15). A recent meta-analyisis of switching clinical trials reinforced this beneficial effect of TAF over TDF (16). In this line, Maggiolo et al. (17, 18) reported an increase in bone mineral density at lumbar or hip sites 48 weeks after switching from TDF to TAF in a population older than 60 years. However, we did not find significant changes in BMD values in our study, probably because they constitute a younger population, with a small sample size and with a shorter period of follow up. Nevertheless, bone mineral density usually takes at least 48 weeks to detect some significant changes. Despite of this, we found significant improvement of bone quality in individuals switching to TAF at 24 weeks of follow up. The increases in bone quality observed in the TAF arm after switching from tenofovir disoproxil fumarate have potentially important clinical consequences in terms of reducing the risk of fragility fractures and its associated morbidity and mortality.

Even though BMD is the gold standard predictor of fragility fractures, incident fractures among HIV individuals are not directly correlated with reduced BMD (4). Therefore, bone quality provides additional information about bone health to BMD and needs to be added to the equation of bone resistance to fracture. Interestingly, we found that the switch to a TAF regimen was associated with a significant improvement of bone quality of 5%. This increase is similar to those observed in naïve HIV individuals that start ABC-3TC-based regimen (10) or TDF-based regimen (9).

All of data suggests that TDF has a larger impact in bone than the PI, INSTI, or NNRTI.

In both ART-experienced and ART-naive PLWHIV, INSTI demonstrated better bone safety profile. In a randomized clinical trial, raltegravir was found to be linked with reduced bone loss when taken with TDF/FTC compared to either darunavir/ritonavir (r) or atazanavir/r (19). Similarly, after switching from a triple therapy including TDF in virologically suppressed PLWH with low BMD -1.0 T-score at weeks 24 and 48, raltegravir in combination with a boosted PI has also been linked to a significant rise in BMD at both the spine and hip (20). Similar results have been reported with BIC (18) or DTG (10). Consequently, TDF is the most likely responsible of the changes in bone quality reported in this study.

Finally, in this study we found a significant association between switch to TAF and weight gain as previously reported due to the lowering weight effect of TDF. However, this increase was associated with an improvement in bone quality (21–23). It is well-Known that body weight is a significant predictor of bone mineral status (24–26),thereby we cannot rule out that BMI might have a role in the bone quality improvement.

This study has some limitations that must be stated. This is a pilot trial with a reduced sample size in a single center. The reported results must be confirmed in larger studies. However, changes found in this study are physiologically plausible and deserve further studies. Other limitation could be the differences in vitamin D levels between the two timepoints. This could be due to the inclusion took after summer, and the follow up after Winter (24 weeks later) likely reflecting the changes of vitamin D over the year. Despite of that, since this is a randomized study and both arms are balanced in the main variables.

One strength of the study is that microindentation is a now plenty available technique that has been recently approved for clinical use and we, as a group, have wide experience with it. The variability between observations is low and the same investigator performed all the measurements.

In conclusion, we present a longitudinal randomized switch study where individuals under TDF-based regimen change to TAF and bone tissue quality is assessed. We found that TAF-based group experienced an improvement in bone quality 24 weeks after switching from TDF independently of BMD. Therefore, microindentation is a sensitive tool for detecting early bone changes. Consequently, measuring other keystone elements of bone strength such as bone tissue quality also provides additional information and may more accurately assess bone health status.

## Data availability statement

The raw data supporting the conclusions of this article will be made available by the authors, without undue reservation.

## Ethics statement

The studies involving human participants were reviewed and approved by Comité de Etica i del Medicament Parc de Salut Mar. The patients/participants provided their written informed consent to participate in this study.

## Author contributions

RG-F, NG-G and JS-F conceptualized and conducted the study. RG-F, OR-L and NG-G revised methodology and did data analysis. RG-F, OR-L, JS-F, HK, IA-A, EC-R, AG-M contributed with recruitment and data curation. All authors have revised and edited the final manuscript. All authos contributed to the article and approved the submitted version.
